# Using Genetic Code Expansion for Protein Biochemical Studies

**DOI:** 10.3389/fbioe.2020.598577

**Published:** 2020-10-19

**Authors:** Christina Z. Chung, Kazuaki Amikura, Dieter Söll

**Affiliations:** ^1^Department of Molecular Biophysics and Biochemistry, Yale University, New Haven, CT, United States; ^2^Department of Chemistry, Yale University, New Haven, CT, United States

**Keywords:** genetic code expansion, non-canonical amino acids, protein labeling, protein purification, protein–protein interactions

## Abstract

Protein identification has gone beyond simply using protein/peptide tags and labeling canonical amino acids. Genetic code expansion has allowed residue- or site-specific incorporation of non-canonical amino acids into proteins. By taking advantage of the unique properties of non-canonical amino acids, we can identify spatiotemporal-specific protein states within living cells. Insertion of more than one non-canonical amino acid allows for selective labeling that can aid in the identification of weak or transient protein–protein interactions. This review will discuss recent studies applying genetic code expansion for protein labeling and identifying protein–protein interactions and offer considerations for future work in expanding genetic code expansion methods.

## Introduction

Heterogeneous environments exist within living cells and this ever-changing environment affects the expression, dynamics, and state of proteins. However, the results and importance of the internal cellular changes are still unclear. Here, we introduce recent progress to observe the internal conditions in living cells using genetic code expansion (GCE).

**SCHEME 1:**
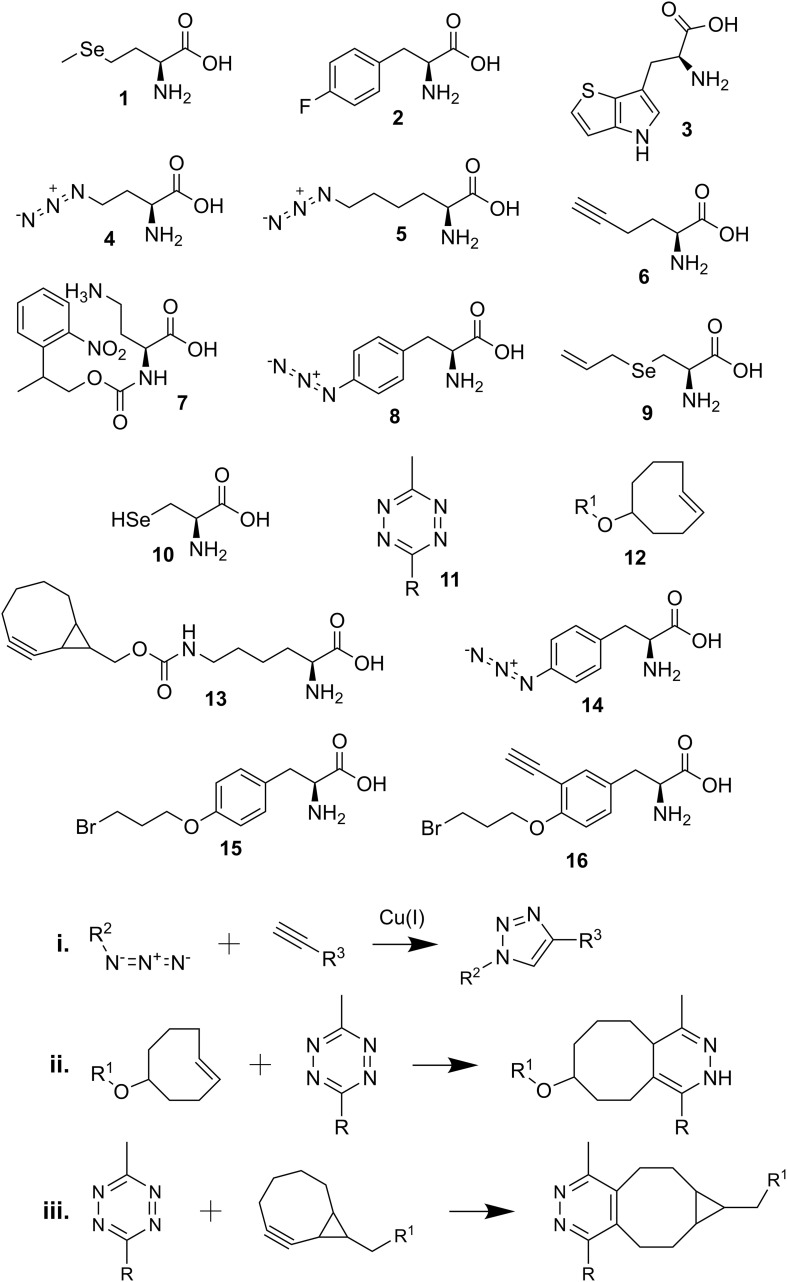
Chemical structures and reactions described in the review. **(1)** selenomethionine; **(2)** p-fluorophenylalanine; **(3)** L-β-(thieno[3,2-b]pyrrolyl)alanine; **(4)** azidohomoalanine (Aha); **(5)** azidonorleucine (Anl); **(6)** homopropargylglycine (HPG); **(7)** photocaged Aha (2-(2-nitrophenyl)propyl Aha); **(8)** p-azido-L-phenylalanine (Azf); **(9)** Se-allyl selenocysteine (ASec); **(10)** selenocysteine (Sec); **(11)** tetrazine-linked dye (R); **(12)** trans-cyclooct-2-ene (TCO^∗^)-linked amino acid (R^1^); **(13)** bicyclo-non-yne lysine (BCN-Lys); **(14)** p-azidophenylalanine (Azi); **(15)** BprY; **(16)** EB3; **(i)** copper-catalyzed alkyne-azide reaction; **(ii)** tetrazine and TCO^∗^ reaction; **(iii)** tetrazine and BCN-linked amino acid reaction.

Proteins are commonly labeled with fluorescent or chemical/light activated molecules *in vitro* and *in vivo* for purposes such as protein purification, determining cellular localization, and visualizing and identifying interacting proteins (Jing and Cornish, 2011; Toseland, 2013; Lang and Chin, 2014; Bartoschik et al., 2018; Chaganti et al., 2018). Using GCE to insert non-canonical amino acids (ncAAs) in proteins of interest offers a high degree of specificity for downstream labeling *in vitro* and *in vivo*. All ncAAs structures and reactions mentioned in this paper are listed in Scheme 1. The incorporation of ncAAs into cellular proteins can be achieved in either a residue- or site-specific manner through protein translation. Traditionally, the incorporation of ncAAs has been accomplished with structurally similar amino acid analogs via selective pressure incorporation into an auxotrophic host (SPI). This labeling strategy allows easy incorporation of ncAAs into proteins without any genetic manipulation with endogenous tRNAs and aminoacyl-tRNA synthetases (aaRSs) (Budisa, 2004). One canonical amino acid in all cellular proteins is replaced with its derivative, e.g., methionine with selenomethionine (1) or phenylalanine with p-fluorophenylalanine (2) (Cowie and Cohen, 1957; Munier and Cohen, 1959). This strategy even led to the complete proteomic replacement of 20,899 Trp residues with its analog L-β-(thieno[3,2-b]pyrrolyl)alanine (3) in *Escherichia coli* (Hoesl et al., 2015). SPI has allowed the incorporation of dozens of ncAAs into cellular proteins and can be used for multiple investigative purposes, such as protein purification, determining cellular localization, and identifying interacting proteins (Lang and Chin, 2014). In a recent report, utilizing a photocaged ncAA provided additional control over protein translation (Adelmund et al., 2018) or even optical control of protein bioadhesion properties (Hauf et al., 2017).

The site-specific incorporation of ncAAs using orthogonal aaRS/tRNA pairs allows observation of a specific protein (Lang and Chin, 2014; Saleh et al., 2019b). However, this method is applicable only to genetically manipulatable organisms where the aaRS/tRNA pairs are orthogonal. In recent years, progress has been made to create aaRS/tRNA pairs that are orthogonal in more organisms (Gohil et al., 2020), including multicellular organisms such as *Caenorhabditis elegans*, *Drosophila melanogaster*, *Danio rerio*, and *Mus musculus* (Greiss and Chin, 2011; Bianco et al., 2012; Ernst et al., 2016; Chen et al., 2017). Stop and sense codon suppression is commonly used for site-specific incorporation of ncAAs. Although these techniques lead to stochastic incorporation of ncAAs, they are hindered by competition with intrinsic factors such as release factors and native tRNAs. Efforts in the past decade attempt to address this problem by reassigning rare codons to encode ncAAs through genome engineering and synthesis (reviewed in Mukai et al., 2017). Previous studies have accomplished this by eliminating the specific assignment of the amber stop codon to signify translation termination or by reassigning rare arginine codons (AGG, AGA) to encode ncAAs (Mukai et al., 2015a,b; Wang and Tsao, 2016). In a recent study, two sense codons (UCG, UCA) and the amber stop codon in the synthetic *E. coli* MDS42 strain were completely eliminated for GCE (Fredens et al., 2019). Various reports have utilized cell-free protein synthesis and *in vitro* transcribed tRNA sets to successfully eliminate or reassign rare codons to encode ncAAs as well as to swap codons for canonical amino acids (Iwane et al., 2016; Cui et al., 2017; Fujino et al., 2020; Hibi et al., 2020). These engineered strains and techniques will contribute to the observation of protein dynamics and the development of protein biochemical studies. This review will present recent studies that develop and utilize various GCE methods to study the cellular dynamics of protein expression and interactions.

## Purification of Newly Synthesized Proteins

**FIGURE 1:**
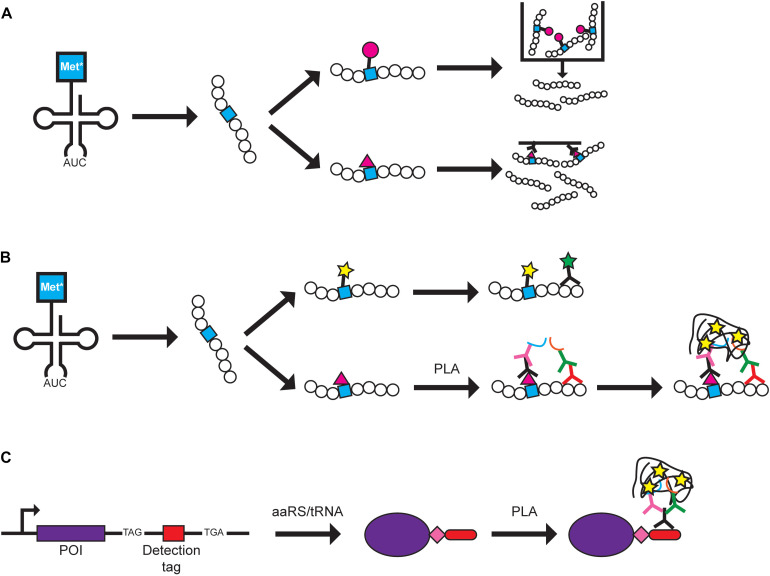
Protein labeling methods using GCE. Examples of how BONCAT and FUNCAT can be used are shown. A Met analog (Met*) is charged onto tRNA^*Met*^ by MetRS and is incorporated at Met codons. **(A)** In BONCAT, Met* can be directly conjugated to azide or alkyne-conjugated beads (pink circle) for purification (top pathway) or conjugated to azide or alkyne-conjugated biotin (pink triangle) for affinity purification (bottom pathway). **(B)** In FUNCAT, Met* can be fluorescently labeled via click chemistry and visualization of a specific protein can be achieved using a fluorescent antibody (top pathway). Alternatively, proximity ligation assay (PLA) can be performed by conjugating Met* to azide- or alkyne-conjugated biotin followed by addition of primary antibodies recognizing biotin and a specific protein. Separate secondary antibodies conjugated to DNA oligonucleotides (blue and orange lines) allow DNA rolling circle amplification that can be targeted by fluorescently labeled complementary probes for signal amplification (bottom pathway). **(C)** In SCROL, a labeling cassette containing a TAG stop codon, a detection tag (e.g., HA-tag and mCHERRY), and an alternative stop codon (e.g., TGA) are genomically incorporated at the 3′-end of the protein of interest (POI). Transfection of an orthogonal aaRS/tRNA pair and ncAA (pink diamond) produces a tagged protein that can be detected using PLA where a primary antibody targets the tag and two secondary antibodies target different parts of the primary antibody.

Metabolic labeling allows cellular protein synthesis to be studied under different environmental or metabolic conditions of cell growth (Beynon and Pratt, 2005). Bioorthogonal ncAA tagging (BONCAT) was developed to label newly synthesized proteins in mammalian cells for purifications and identification (Dieterich et al., 2006, 2007) (Figure 1A). Azidohomoalanine (Aha) (4), a methionine (Met) analog, is charged onto tRNA^*Met*^ and incorporated into newly synthesized proteins at Met codons. The azide group of Aha is used to selectively purify the proteins produced within a desired timeframe during growth of mammalian cells. Tagged proteins can subsequently be identified through mass spectroscopy (Dieterich et al., 2006). Moreover, azidonorleucine (Anl) (5) has been used in various cell-type specific labeling studies. This azide-containing Met analog has been demonstrated to be charged onto initiator tRNA^*Met*^_*i*_ by a mutant methionyl-tRNA synthetase (MetRS) from *E. coli* (Ngo et al., 2013) and onto elongator tRNA^*Met*^ by a mutant murine MetRS (Mahdavi et al., 2016) in mammalian cells for identification of newly synthesized proteins. In both studies, only expression of the mutant MetRS is required to incorporate Anl and cell-specific labeling can be achieved via Cre-induced expression of mutant MetRS (Erdmann et al., 2015; Alvarez-Castelao et al., 2017, 2019; Evans et al., 2020). Alternatively, the alkyne-bearing amino acid homopropargylglycine (HPG) (6) has been suggested as a different Met analog for affinity purification (Landgraf et al., 2015).

While BONCAT was developed in mammalian cell cultures (Dieterich et al., 2006), the method can be adapted for other systems. The labeling method has been combined with Cre-induced MetRS expression for cell-specific labeling in mice (Alvarez-Castelao et al., 2017) and used to study protein synthesis in neurodegenerative disease mouse models (Evans et al., 2019) and during mouse and zebrafish development (Hinz et al., 2012; Saleh et al., 2019a). In each of those studies, the authors were able to identify proteins within specific timeframes and observe changes in the proteome under different conditions. In the plant *Arabidopsis thaliana*, BONCAT was used to isolate and identify proteins expressed under different conditions of stress via click chemistry to dibenzocyclooctyne beads and mass spectrometry. The authors were able to demonstrate that stress-related proteins were increased upon stress induction (Glenn et al., 2017).

Light-activated BONCAT is a modified version of BONCAT where photocaged Aha (7) can only be incorporated into proteins upon uncaging by light exposure. The amount of protein labeling in HeLa cells could be controlled by varying the light intensity and proteins in specific regions can be labeled by aiming the light source, allowing spatial and temporal control of Aha incorporation (Adelmund et al., 2018). However, proteins that do not encode at least one Met codon within their sequence will not be detected, as the N-terminal Met is often cleaved off (Wingfield, 2017). This can be circumvented by utilizing a different amino acid analog, such as the insertion of p-azido-L-phenylalanine (Azf) (8) at Phe codons in *C. elegans* by a mutant *C. elegans* phenylalanyl-tRNA synthetase (Yuet et al., 2015). Nevertheless, BONCAT provides a snapshot of proteins that are produced under various conditions. This can lead to the discovery of proteins that are only produced under specific conditions, and help researchers understand how proteins can play multiple roles.

## Protein Visualization Using Fluorescent Molecules

Genetic code expansion has allowed the ability to selectively label proteins in living cells by taking advantage of the unique properties of the encoded ncAA. A protected selenocysteine (Sec) (9) was site-specifically incorporated in the outer membrane protein eCPX (enhanced circularly permuted outer membrane protein OmpX) of *E. coli* by recoding the UAG stop codon (Liu et al., 2018). By taking advantage of the different pKa values of cysteine (pKa 8.3) and Sec (pKa ∼5.2) (10), selective labeling of the chemically deprotected Sec with a fluorescent dye through thiol chemistry was achieved at pH 5.0 (Liu et al., 2018).

To visualize the spatial localization of newly synthesized proteins, BONCAT was modified to visualize proteins and termed fluorescence non-canonical amino acid tagging (FUNCAT) (Dieterich et al., 2010). Fixed cells from mammalian cell cultures and mouse models with newly synthesized proteins containing Aha or HPG are tagged with fluorescent dyes via click chemistry or can be combined with immunochemistry to visualize specific proteins (Dieterich et al., 2010; Hinz et al., 2012; Evans et al., 2019) (Figure 1B). FUNCAT has also been coupled with the proximity ligation assay (PLA) to visualize the spatial localization of specific proteins in fixed cells (tom Dieck et al., 2015). Primary antibodies recognize either the Aha-tagged protein or a specific protein and each primary antibody is recognized by a secondary antibody that carries different DNA adaptors. The adaptors are used for rolling circle amplification and the resulting DNA product is labeled with complementary fluorescently labeled probes, producing an amplified signal for each target protein molecule (tom Dieck et al., 2015). The authors noted that although it is possible that the PLA signal can result from two closely positioned proteins, it is more likely that both primary antibodies bind to the newly synthesized target protein to produce the PLA signal. Using Met analogs to label newly synthesized proteins has expanded to studying bacteria and microbes in environmental and human samples. The fluorescently labeled Met analog can be combined with other techniques such as FISH or FACS to study the translationally active cell populations (Couradeau et al., 2019; Sebastian and Gasol, 2019; Valentini et al., 2020). Thus, labeling of the Met analog shows promise as a tool that can be widely used in different biological systems in combination with other visualization techniques.

In an effort to further improve upon the specificity of FUNCAT-PLA, SCROL (Stop-Codon-Read-thrOugh-Label) was developed to study expression of a specific protein of interest (Schneider et al., 2018) (Figure 1C). A cassette containing the UAG amber codon, HA-tag, and an alternative stop codon (e.g., UGA opal codon) was genomically inserted at the 3’-end of the target protein using CRISPR/Cas9 in HEK293 cells. It is important to note that, even in the presence of an orthogonal aaRS/tRNA pair, the tagged protein will only be expressed upon addition of the ncAA, and thus, the protein can be labeled at specific timeframes. This would ensure that the cell, and protein of interest, is undisturbed in the absence of the ncAA. In this study, the ncAA was not used for labeling/detecting the proteins but as a method to control expression of the epitope tag. This differs from many other papers where the purpose of incorporating an ncAA is to directly use it in future studies. Thus, additional specialized aaRS/tRNA pairs and ncAAs would not need to be developed for this technique.

An important component of labeling ncAAs *in vivo* for protein visualization under different cellular conditions is bioorthogonal dye. Tetrazine dyes (11) have been extensively characterized for their use in labeling trans-cyclooct-2-ene (TCO^∗^) modified ncAAs (12) via click chemistry in a variety of mammalian cells for live cell imaging (Beliu et al., 2019). An attractive trait of the dyes is their small size compared to traditional labeling molecules such as antibodies. By choosing cell membrane-permeable or impermeable dyes based on the target protein, selective labeling can be enhanced. The authors demonstrated selective and efficient labeling of the target proteins that produced superior readings compared to immunolabeling (Beliu et al., 2019). A similar study utilized bicyclo-non-yne lysine (BCN-Lys) (13) to attach a tetrazine dye to the protein of interest via a N-terminal tag in HEK293T and Cos7 cells (Segal et al., 2020). Interestingly, insertion of BCN-Lys in the N-terminal tag was labeled better than BCN insertion within the target protein. In addition, the modified tag was found to be compatible in labeling organelle proteins within their acidic environments. Although the authors demonstrated the ability to switch out the HA-tag with other commonly used tags (Myc and FLAG), the results were not as successful, indicating a need for further optimization (Segal et al., 2020). The ease of simply inserting a tag encoding an ncAA led the authors to state the possibility of genomically inserting the modified tag to monitor endogenously expressed proteins. This would ensure that any effects on protein folding and interactions would be minimized and the use of the tetrazine dyes allows live-cell imaging. Live-cell imaging using fluorescently labeled proteins provides a story of how proteins travel and react within the cell under various conditions, rather than a picture obtained from fixed cells. This could be helpful in studying disease-associated proteins and understanding how cellular and environmental changes affect their spatial distribution.

Insertion of different ncAAs within a single protein allows different molecules to be attached, permitting the application of various techniques. However, the limited number of available codons for reassignment/recoding, orthogonal aaRS/tRNA systems, and orthogonal labeling reactions impairs the actual number of insertable ncAAs. Recently, three different ncAAs were inserted within a single protein using three different orthogonal aaRS/tRNA pairs in *E. coli* (Italia et al., 2019). The same group had previously engineered an *E. coli* strain (ATMW1) where the endogenous tryptophanyl–tRNA synthetase/tRNA^*Trp*^ (TrpRS/tRNA^*Trp*^) pair was replaced by the TrpRS/tRNA^*Trp*^ from *Saccharomyces cerevisiae*, rendering the *E. coli* TrpRS/tRNA^*Trp*^ pair available for ncAA insertion (Italia et al., 2017). As three distinct ncAAs were inserted using all three nonsense suppressors, homogenous translation termination was achieved by inserting a TEV cleavage site at the C-terminal end of sfGFP-His followed by TEV protease and 3 consecutive UAA stop codons and all three ncAAs could be labeled in a one-pot reaction (Italia et al., 2019). More recently, two separate classes of pyrrolysyl-tRNA synthetase (PylRS) with the N-terminal domain missing and their corresponding tRNAs were identified to be mutually orthogonal to each other and to a *Methanosarcina mazei* PylRS/*Spe*tRNA^*Pyl*^ pair in *E coli*. By co-expressing all three pairs and ribo-Q1 with a GFP reporter, three distinct ncAAs were incorporated by recoding the UAG stop codon and two quadruplet codons (Dunkelmann et al., 2020). The use of quadruplet codons would allow protein termination with a triplet stop codon. Alternatively, competitive labeling of one ncAA reserves a nonsense codon for translation termination while still allowing production of the multi-labeled protein for different studies (Konig et al., 2020). By taking advantage of the ability of BCN-Lys to be labeled by various tetrazine-conjugated fluorescent dyes via click chemistry, two different dyes could be conjugated simultaneously to allow the use of two different techniques that require different fluorescent parameters. This was demonstrated to be feasible in live Cos7 cells, allowing real-time high resolution imaging at a single molecule level (Konig et al., 2020). These studies demonstrate the ability to insert multiple ncAAs within a single protein where each ncAA can take part in a specific chemical reaction. This would greatly increase the yield of samples that require multiple downstream processing steps as constant protein purification for different labeling reactions would not be required.

## Identifying Protein–Protein Interactions

Studying the interactions of proteins aids in the understanding of their cellular roles under normal and stress conditions. Co-immunoprecipitation, crosslinking, and BioID are some techniques used to identify interacting proteins (Lin and Lai, 2017; Yu and Huang, 2018; Sears et al., 2019). GCE can be used to insert crosslinking ncAAs at known or potential binding interfaces to increase the chances of identifying interacting proteins as well as minimize interference with protein folding and activity. The light-activated crosslinker p-azidophenylalanine (Azi) (14) was demonstrated in *E. coli* to be efficiently incorporated and crosslinks with amines of interacting proteins (Chin et al., 2002). Recently, Azi was successfully inserted at recoded UAG stop codons and used to map protein-protein interactions within the inner membrane complex of the protozoan *Toxoplasma gondii* (Choi et al., 2019). Chemical crosslinkers are an alternative to light-activated crosslinkers. Two chemical crosslinkers have been developed that spontaneously react with cysteine residues that are in close proximity: BprY (15) and EB3 (16). Both crosslinkers are alkyl bromides with EB3 containing an alkyne group for enrichment with biotin. Their applicability in detecting weak and transient interactions was demonstrated in live *E. coli* cells (Yang et al., 2017). The drawback of these chemical crosslinkers is the assumption that cysteine residues are present at the binding interface. Replicating experiments with different types of crosslinkers could increase the coverage of potential interacting proteins. Using genetically encoded crosslinkers offers a high degree of specificity that can capture strong and transient interactions.

## Discussion

The studies discussed in this review demonstrate the potential of utilizing GCE to label proteins in an effort to illuminate the heterogenous intracellular environment. We present the following ideas for consideration to improve the current methods. (1) Although aaRS/tRNA pairs are tested to ensure their orthogonality within a system, cross-reactions may still occur. Developing more specific aaRS/tRNA pairs that minimally cross-react with endogenous aaRSs and tRNAs would lessen the impact on cellular metabolism and allow a more “natural” representation of the protein(s) of interest due to minimal disturbance of the intracellular environment. In addition, there is a need to produce more aaRS/tRNA pairs that are orthogonal in a greater variety of organisms (Greiss and Chin, 2011; Bianco et al., 2012; Ernst et al., 2016; Chen et al., 2017; Gohil et al., 2020) to study proteins in their natural environment with any necessary post-translational modifications that are often missing when produced in model expression organisms such as *E. coli*. (2) Development of more sensitive detection techniques would enable a greater understanding of localization for more proteins. Although FUNCAT coupled to PLA allows amplification of a signal for a protein of interest (tom Dieck et al., 2015), a high affinity antibody must be available. This is often not the case for many proteins where only low affinity antibodies are available or none have so far been produced. While SCROL offers an alternative where the peptide tag is targeted by an antibody (Schneider et al., 2018), the SCROL cassette must be genomically incorporated to produce a stable cell line. (3) The increasing number of ncAAs allows for the development and optimization of techniques for specific purposes. However, this is often hindered by the limited quantity and price of ncAAs. Many ncAAs are expensive to buy or to chemically synthesize within the lab. It would be beneficial to develop a method to produce greater quantities of ncAAs, biologically or chemically. A related issue is the development of ncAAs for organelle-specific labeling. These ncAAs must be stable in the organellar environment and chemically reactive ncAAs would have to be designed to react specifically within the organellar environment. Light-activated ncAAs would circumvent this issue and would likely offer greater control in activating the ncAA.

## Conclusion

In conclusion, GCE has become a versatile tool that can be modified to suit individual needs and incorporated into existing techniques. In the future, it is expected that more tools will be developed for expansion of GCE to other organisms and to improve on existing GCE methods for understanding heterogeneous environments within living cells.

## Author Contributions

CZC and KA wrote the manuscript. DS edited it. All authors contributed to the article and approved the submitted version.

## Conflict of Interest

The authors declare that the research was conducted in the absence of any commercial or financial relationships that could be construed as a potential conflict of interest.
